# Development of the circadian system in early life: maternal and environmental factors

**DOI:** 10.1186/s40101-022-00294-0

**Published:** 2022-05-16

**Authors:** Sachi D. Wong, Kenneth P. Wright, Robert L. Spencer, Céline Vetter, Laurel M. Hicks, Oskar G. Jenni, Monique K. LeBourgeois

**Affiliations:** 1 Department of Integrative Physiology, University of Colorado Boulder, 354 UCB, Boulder, CO 80309 USA; 2 Department of Psychology and Neuroscience, University of Colorado Boulder, Boulder, CO USA; 3 Renée Crown Wellness Institute, University of Colorado Boulder, Boulder, CO USA; 4 Child Development Center, University Children’s Hospital Zurich, University of Zurich, Zürich, Switzerland

**Keywords:** Circadian, Development, Light, Sleep, Circadian rhythm, Fetus, Neonate, Melatonin, Cortisol, Rest-activity

## Abstract

In humans, an adaptable internal biological system generates circadian rhythms that maintain synchronicity of behavior and physiology with the changing demands of the 24-h environment. Development of the circadian system begins in utero and continues throughout the first few years of life. Maturation of the clock can be measured through sleep/wake patterns and hormone secretion. Circadian rhythms, by definition, can persist in the absence of environmental input; however, their ability to adjust to external time cues is vital for adaptation and entrainment to the environment. The significance of these external factors that influence the emergence of a stable circadian clock in the first years of life remain poorly understood. Infants raised in our post-modern world face adverse external circadian signals, such as artificial light and mistimed hormonal cues via breast milk, which may increase interference with the physiological mechanisms that promote circadian synchronization. This review describes the very early developmental stages of the clock and common circadian misalignment scenarios that make the developing circadian system more susceptible to conflicting time cues and temporal disorder between the maternal, fetal, infant, and peripheral clocks.

## Introduction

The internal circadian clock of humans governs daily physiological functions, including the sleep/wake cycle and rhythms of the hormones melatonin and cortisol [1, 2]. In healthy adults and children, the central biological clock adheres to a 24-h cycle by entraining to environmental time cues, the strongest being light. Circadian disruption and dysregulation in adults are associated with a number of physical and mental health conditions, including insomnia, neurodegenerative disorders, metabolic disease, and psychiatric disorders [3–7]. Development of the circadian system begins in utero and continues throughout early life. Data from rodent studies indicate that circadian disruption during fetal development is associated with adult-onset cardiovascular disease, reduced bone mass and strength, glucose intolerance, insulin resistance, and mood disorder-related behaviors including anhedonia- and depression-like behaviors [8–12]. In human fetuses and infants, little is known about the links between circadian system maturation and health, as well as the factors that influence the development of an adaptive biological clock.

Sleep disruption during infancy is prevalent and persistent. Cross-sectional data show that 10–46% of infants ages 6–18 months exhibit some type of sleep disturbance (e.g., bedtime fussiness, difficulty falling asleep, frequent, and prolonged night awakenings) [13–17]. Persistence of infant sleep problems through the preschool years is common and can track into late childhood and adolescence [15, 17]. Results from previous studies indicate a relationship between parent-reported sleep difficulties and child psychosocial issues, including aggressive behavior, attention problems, anxiety, hyperactivity, and mood problems [17–22]. Additionally, lower cognitive performance and poor school functioning are associated with childhood sleep problems, suggesting that sleep issues may play a role in diminishing cognitive processes [23–25]. On the familial level, infant sleep problems are associated with higher maternal depression and poorer global health in both parents [14, 17, 26]. Identifying modifiable risk factors at the earliest stage of development provides an important opportunity to understand how to prevent and remedy infant sleep problems and related health conditions, both concurrent and those that emerge in the future.

**Fig. 1:**
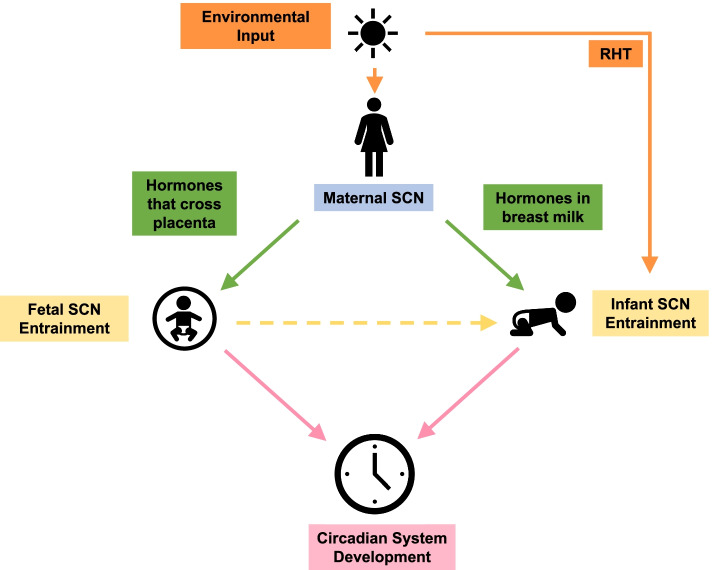
Model for circadian system development

The prevalence of infant sleep problems may be due to misalignment between environmental inputs and endogenous biological rhythms across the prenatal and post-natal periods that influence development of the circadian system. Human fetuses and infants mature in the context of an artificial light-saturated environment, and the acute and chronic effects of this circadian misalignment on development remain largely unknown. This comprehensive review will describe what is known about human circadian rhythms in fetal and infant development, as well as factors that influence sleep and the circadian system in early life. It will (1) discuss the two-process model of sleep regulation, a foundational framework for understanding the interaction between sleep homeostasis and the circadian rhythm; (2) review the fundaments of circadian physiology; and (3) present different proposed mechanisms underlying development of the circadian system (Fig. 1).

Light is the strongest zeitgeber to the maternal suprachiasmatic nucleus (SCN) and regulates the diurnal fluctuation of maternal hormonal time cues directly to the fetus through the placenta and indirectly to the infant through breast milk. Additionally, light entrains the infant SCN via the developing retinohypothalamic tract (RHT). These environmental and maternal signals interact with the fetus and infant to influence the development of the circadian system.

## Two-process model of sleep regulation

As first proposed by Borbély in 1982 [27], sleep regulation is governed by the interaction between a clock-dependent circadian process, Process C, and a sleep/wake dependent homeostatic process, Process S. Process C oscillates as an approximate 24-h rhythm, with the drive for sleep beginning in the evening and reaching its peak in the middle of the night, followed by a drive for wakefulness that rises in the early morning [28]. Process S is determined by the amount of prior wakefulness, such that the longer an individual is awake, the greater the “pressure” to sleep. Sleep pressure builds in an exponential fashion across the day and dissipates throughout time spent sleeping [29]. These two processes are independent but interact to control levels of alertness and sleep drive. As sleep pressure rises throughout the day, it is opposed by a stronger circadian drive for wakefulness. At bedtime, sleep propensity is promoted by the coordination of melatonin secretion and a high level of sleep pressure [30]. Melatonin is synthesized and released by the pineal gland and binds to MT1 and MT2 receptors in the suprachiasmatic nucleus (SCN) to help synchronize the circadian rhythm [31]. As sleep unfolds across the night, sleep pressure dissipates; however, the increase in the circadian promotion of sleep results in consolidation until waking in the morning. The synergy of these two systems is essential to promote optimal sleep at night and wakefulness during the day, and misalignment results in reduced sleep quality and duration [32–35].

## Infant sleep

**Fig. 2:**
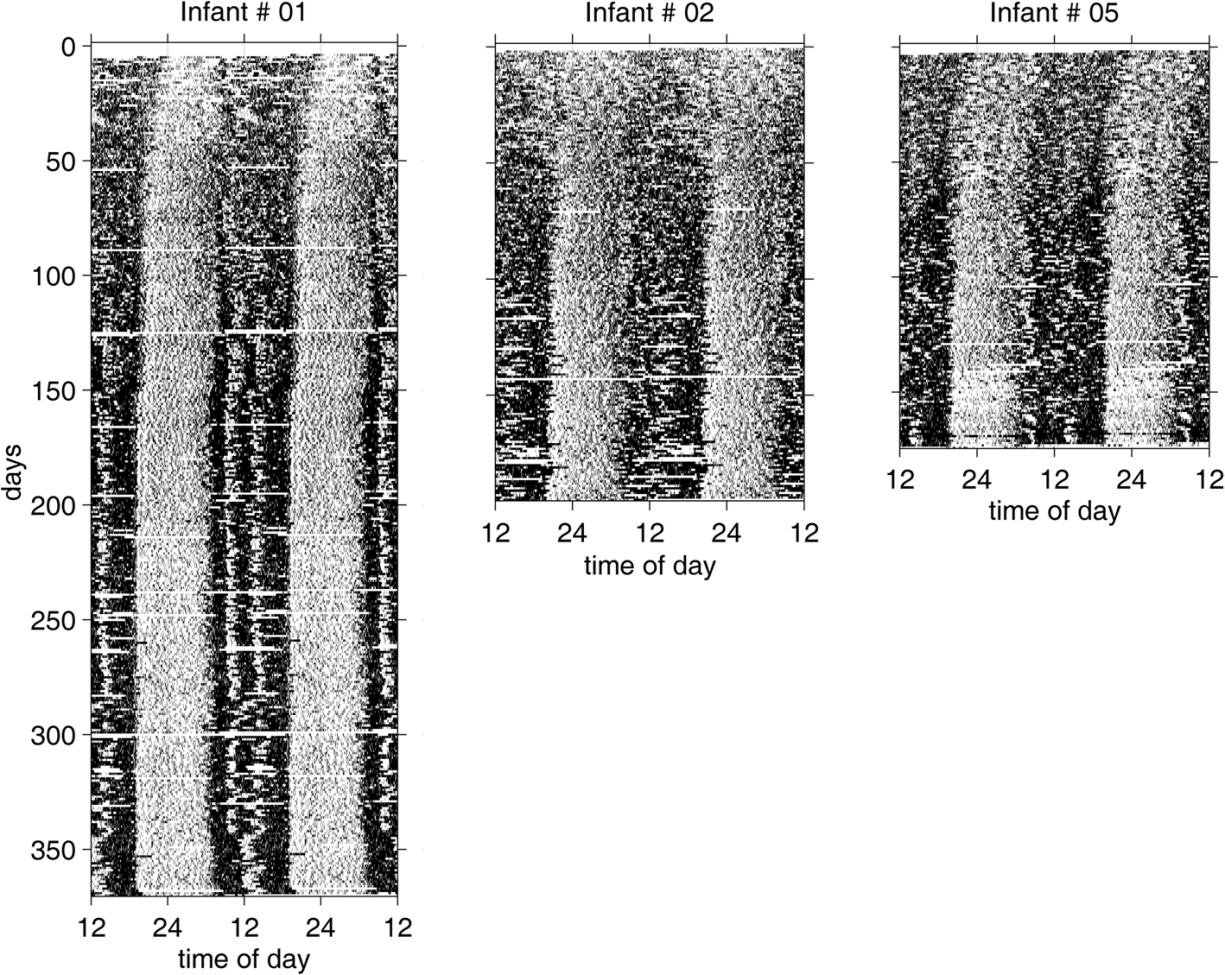
Development of rest-activity patterns in infants

Newborns spend about 70% of their first few weeks after birth sleeping, and the timing of their sleep episodes is distributed equally throughout the 24-h day with no clear rhythm [36, 37]. At 2 weeks of age, infants sleep in approximately 4-h intervals [38]. As measured by actigraphy, Jenni and colleagues [39] observed day-night differences in rest-activity patterns within the first weeks of infancy (Fig. 2). By 5 weeks of age, there was emergence of an infradian rhythm, with a 25-h period as circadian rhythmicity began to appear [36]. At about 15 weeks, more consolidated wake and sleep episodes were apparent, and by 6 to 9 months of age most infants were able to sleep through the night, displaying at least 6-h consolidated sleep episodes [40–42]. Average total sleep duration across the first year of life remained about 14 h per day. However, it was the lengthening and timing of sleep and wake episodes, an increase in sleep during the night and an increase in wakefulness during the day, that matured with development. In other words, older infants, with more mature circadian regulation, were more likely to sleep during the night for longer durations than newborns. It should be noted, however, that alongside circadian system development, maturation of the sleep homeostatic process also plays a role in the emergence of this observed 24-h pattern in the first few months of life [40, 43]. Additionally, individual variability of sleep timing and duration is large [42], representing differences in the developmental manifestation of sleep-wake and circadian synchronicity.

Double plotted rest-activity patterns of three infants. The horizontal axis represents 48 h, black areas represent activity, and white areas depict rest episodes. Data were continuously collected in 1-min epochs via actigraphy over 6 to 12 months. Initially, post-birth, infants had weak day-night asymmetry, with sleep and wake occurring at all hours of the 24-h day. Infants 1 and 5 developed an ultradian pattern of rest and activity that emerged in the first few weeks of life, followed by more nighttime sleep consolidation and a regular daytime nap schedule. Infant 2 displayed a less robust ultradian rhythm in the first few weeks and more irregular daytime naps. These three infants demonstrate the interindividual variability in rest-activity patterns observed in early development. Note: Reprinted from “Development of the 24-h rest-activity pattern in human infants,” by O. G. Jenni, T. Doboer, and P. Achermann, 2006, *Infant Behavior & Development*, *29*, p. 143–152. Copyright 2005 by Elsevier Inc. Adapted with permission.

## Circadian fundaments

Circadian (Latin for “circa diem,” meaning “around a day”) rhythms are endogenous biological rhythms within organisms that oscillate in the absence of environmental time cues, entrain to environmental signals, and remain stable in environments with fluctuating temperatures [44]. For biological processes to occur at the appropriate time in the proper sequence, organisms must be able to synchronize to and predict their external environment. This ‘temporal framework’ allows organisms to anticipate daily changes and optimize their behavior and physiology in advance of the daily demands of the light-dark cycle [1].

At the molecular level, the SCN maintains 24-h rhythmicity through positive and negative transcriptional-translational feedback loops of clock genes [45]. In mammals, the positive feedback loop is initiated during the biological day through positive regulating genes Bmal1 (Brain and muscle Arnt-like factor) and Clock (Circadian locomotor output cycle kaput) and their protein products [1, 46]. Within the nucleus, proteins BMAL1 and CLOCK heterodimerize and bind to E-box response elements within promoter regions of negative regulating genes Per (Per1 and Per2) and Cry (Cry1 and Cry2), inducing their transcription. Per and Cry mRNA diffuse into the cytoplasm where they are translated into PER and CRY proteins by ribosomes. At night, PER and CRY proteins in association with casein kinase 1 and other proteins translocate back into the nucleus with the help of GAPVD1, a cytoplasmic trafficking factor, where they inhibit CLOCK:BMAL1 transcriptional activity and subsequently close the negative feedback loop [47]. Casein kinase 1 also mediates proteasome degradation of PER through phosphorylation, thus lifting the inhibition of BMAL1 and CLOCK and allowing a new cycle of positive transcriptional activity to commence [48]. The CLOCK:BMAL1 complex also drives the transcription of Rorα (retinoic acid receptor-related orphan receptor α) and Rev-Erbα (reverse erythroblastosis virus α) genes, which serve as activators and inhibitors of Bmal1 transcription respectively [45]. This second feedback loop in conjunction with the previously described loop is responsible for a robust 24-h oscillatory molecular circadian cycle.

Although the mammalian molecular clock was first identified in the SCN in the late 1990s, subsequently the presence of an operational molecular clock has been observed in many tissues (“peripheral oscillators”) throughout the body [1]. Importantly, however, the circadian system is organized in a hierarchal fashion, with the SCN of the anterior hypothalamus serving as the master clock that works to synchronize peripheral oscillators [49]. SCN lesioned rodents lack circadian activity rhythms, and implantation of new SCN neural grafts is sufficient to restore circadian expression with a period length relative to the new SCN [50]. Thus, the SCN is the central driver of overt circadian rhythms.

In mammals, the SCN is comprised of a dorsal shell of neurons that express the neuropeptide arginine vasopressin (AVP) surrounding a ventral core composed of vasoactive intestinal polypeptide (VIP) expressing neurons [51]. When placed in culture, in vitro SCN neurons maintained a robust 24-h rhythm of Per1 clock gene expression for more than 30 days [52]. Conversely, peripheral tissues that underwent the same treatment only retained Per1 rhythmicity for less than a week, suggesting a loss of synchronization in the absence of rhythmic signals from the SCN. Furthermore, when rats experienced a 6-h delay or advance in their light:dark (LD) 12 h:12 h cycle, their SCN neurons in vitro shifted rapidly in response to environmental light, while peripheral cells had a less adaptive response, indicating that these damped oscillators required an external circadian signal for both rhythmicity and adjustment [52]. These peripheral oscillators are subordinate to the SCN and have been demonstrated in vivo in many tissues including various brain regions [53–55]. It is noteworthy that the phase of oscillatory clock gene expression can vary between different peripheral oscillators and the SCN [56–58]. Therefore, one role of peripheral oscillators is likely to transduce a daily environmentally entrained timing signal from the SCN into the appropriate molecular oscillatory phase for optimal daily function of each tissue [59].

Light is the strongest zeitgeber (“time giver”) of the circadian system. Because the average SCN oscillation in humans is greater than 24 h (approximately 24.15 h), a daily reset is necessary to prevent the endogenous oscillation from drifting too far from the environmental light:dark cycle [60]. Through the retinohypothalamic tract (RHT), a neuronal pathway from the retina to the SCN, the SCN receives photic information that enables it to entrain to the light-dark cycle. This neuronal pathway consists of a special subset of retinal ganglion cells (intrinsic photosensitive retinal ganglion cells) that are exquisitely sensitive to low light levels, especially in the blue portion of the visible spectrum [61]. The SCN transmits this perceived time-of-day information via neural and hormonal signals to peripheral clocks located in brain areas outside of the SCN, as well as to most organs including the pancreas, liver, kidney, and heart [1, 56, 62–64]. The coordination of the SCN with its peripheral oscillators results in necessary and time-of-day appropriate physiological and behavioral outcomes [65]. It should be noted, however, that RHT stimulation of the SCN in response to light at night triggers a phase shift in SCN oscillation as the SCN adjusts its timing to an unexpected environmental light:dark cycle stimulus [66]. This inappropriate phase adjustment provides a major source of circadian disruption to the body.

## Circadian phase markers

The physiology of the SCN cannot be directly assessed in humans, and therefore studying output rhythms of the clock is necessary to determine the timing of the circadian rhythm. Measurable outputs, known as circadian phase markers, include melatonin, cortisol, and core body temperature. Timing of the internal clock is established through measurements of its period, the time necessary to complete a full oscillation, and phase, the state of an oscillation at a specific point in time [67].

Melatonin secretion is regulated by the SCN via a multi-synaptic pathway: from the SCN, neurons project to the superior cervical ganglion (SCG) and then to the pineal gland, which synthesizes and secretes melatonin [2]. Light exposure during the biological night results in rapid suppression of melatonin through this multi-synaptic pathway [68]. In adult humans, melatonin levels increase in the evening, peak during the middle of the night, and return to low levels in the morning where they remain throughout the day.

Cortisol is secreted from the adrenal glands in response to the pituitary gland releasing adrenocorticotropic hormone (ACTH). The circadian sensitivity of the adrenal gland to ACTH is regulated via a multi-synaptic neural output pathway from the SCN [2]. Cortisol secretion is lowest during the early night, begins to increase several hours before awakening, and peaks in the morning within 30–45 min after awakening. Of note, the cortisol awakening response (CAR) is a separate process that is superimposed on the underlying circadian peak of cortisol secretion [69]. It has been hypothesized that the magnitude of the morning CAR is associated with anticipation of stressors for the upcoming day [70].

Core body temperature peaks during the day and reaches its nadir in the middle of the night, which typically aligns with the sleep midpoint [71, 72]. Data from well-controlled studies indicate that the evening rise in melatonin prompts the nocturnal decrease in core body temperature through distal vasodilation and subsequent heat loss [73, 74].

There is growing support for the feasibility of measuring clock gene expression in human peripheral tissues as a useful circadian phase biomarker. Samples studied for these assessments include white blood cells, skin, oral mucosa, colon cells, adipose tissue, and post-mortem brain tissue [58]. Determining clock gene expression in peripheral tissues provides a method to monitor both central and peripheral clock regulation. Researchers are currently working towards validating gene expression from noninvasive skin samples as a biomarker of molecular clock phase to utilize in the rising field of circadian medicine [75].

## Factors influencing circadian development in utero

### Maternal SCN

The developing fetal circadian system relies solely on the maternal circadian system to set the environment context and relay time-of-day information. During pregnancy, maternal physiology changes to meet the needs of the developing fetus, and some of these adaptations are under circadian control. Pregnant women experience changes in circadian regulated plasma cortisol and melatonin concentration, core body temperature, and metabolism [76]. Additionally, women undergo an earlier shift in their chronotype, or their diurnal preference for sleep time, in the first and second trimesters as exhibited through an earlier sleep onset time [77].

### Fetal SCN and peripheral oscillators

The fetal SCN and organs have been proposed to function as peripheral maternal circadian oscillators that are entrained through signals from the maternal SCN [78]. Similar to an orchestra, the maternal SCN serves as a conductor of the fetal clocks to maintain synchronization to the environment. This hierarchal arrangement produces temporal order similar to that found in the peripheral clocks of the adult human.

Circadian rhythmicity may depend on the maturity of the circadian system. Although clock gene expression is present in the SCN in embryonic mice, it appears that circadian system development is contingent on both the maturation of the central clock as well as the signaling pathways to its peripheral oscillators [79]. Dolatshad and colleagues [80] found that embryonic mouse tissues express circadian regulatory genes that appear rhythmic in vitro but lack the tissue level function to oscillate synchronously in vivo. In other words, the cells can synchronize and produce a tissue level rhythm, but for some reason do not. Animals born more developed may have more functional circadian system pathways, as presence of entrained 24-h rhythms in heart rate, respiratory movements, and hormones have been discovered in sheep, monkey, and human fetuses [81].

The human SCN begins developing in utero, and can be detected through histology by 40% gestation [82]. Additionally, the fetal SCN in late term squirrel monkeys exhibits oscillations of glucose utilization; suggesting it may be functional [83]. Because of limitations in human research, utilizing primate models is a powerful approach for determining the function and independence of the fetal SCN. In the SCN of capuchin monkeys at 90% gestation, robust oscillation of clock genes Bmal1 and Per2, and the MT1 melatonin receptor are apparent; however, the amplitude of these gene expression and receptor oscillations are smaller in the fetal primate SCN compared to the adult primate SCN. Additionally, fetal Bmal1 and Per2 oscillate in an antiphase pattern relative to the adult SCN similar to that found in the peripheral clocks of adult capuchin monkeys [84, 85]. It should be noted that in baboons, peripheral clock gene expression is not antiphasic to SCN clock gene expression; thus, this pattern may not be universal in primates [55]. Nonetheless, these blunted, antiphase oscillations in fetal capuchin monkeys suggest that the fetal SCN may be controlled by the maternal SCN. This was further demonstrated through suppression of primate maternal melatonin which yielded a change in the expression of clock genes in the fetal SCN that was restored through maternal melatonin replacement [84]. Taken together, existing research indicates that the maternal primate SCN communicates with the fetal primate SCN via zeitgebers, such as maternal melatonin, to produce rhythmicity.

Although the fetal SCN does not function independently from the maternal SCN, it may be important for receiving maternal signals and integrating them into physiological outputs. Lunshof and colleagues [86] tested this hypothesis by recording 24-h rhythms in fetal heart rate in a discordant anencephalic twin pregnancy, a disorder that results in an absent cerebellum and SCN. Results showed a significant diurnal rhythm in the mother as well as the intact twin fetus but not in the anencephalic fetus. These findings suggest that the fetal brain, and most likely the fetal SCN, are vital for the generation of circadian rhythms within the developing fetus.

In adult humans, rhesus monkeys, and mice, the adrenal gland serves as a peripheral clock that receives input from the SCN and in turn generates circadian rhythmicity of glucocorticoids [87–89]. Conversely, the fetal adrenal gland likely receives input from the maternal SCN. Torres-Farfan [84] and colleagues found that capuchin monkeys at 90% gestation exhibit circadian gene expression of Bmal1 and Per2, and the MT1 receptor. This temporal pattern of gene oscillation was identical to that of the fetal SCN, indicating that both the fetal adrenal gland and SCN are under similar control. In adult capuchin monkeys, there is a phase delay between peripheral oscillators and the SCN [85], and the absence of this delay between the fetal SCN and adrenal gland suggests that the maternal SCN, not the fetal SCN, regulates the fetal adrenal. However, there may be another integrator necessary to maintain rhythmicity of the fetal adrenal gland. In capuchin monkeys, suppression of the maternal SCN resulted in shifted fetal clock gene expression, but the fetal adrenal gland remained unaffected [84]. The maternal adrenal gland may account for lack of rhythmic change. In humans, suppression of maternal adrenal function with glucocorticoid treatment (triamcinolone) resulted in the disappearance of rhythms in fetal heart rate and limb movements. Rhythms were restored post-treatment, indicating fetal rhythm dependence on the maternal adrenal [90]. These data suggest that the fetal adrenal gland relies on input from both the maternal SCN and adrenal gland and remains independent from the fetal SCN. Further research is needed to examine the hierarchy of maternal signals to which the fetal adrenal entrains to.

### Fetal circadian entrainment through melatonin

In utero, the fetus is reliant on time-of-day signals from maternal circadian cues (e.g., melatonin, cortisol, temperature), which serve as entrainers to the fetal circadian system and peripheral clocks. One zeitgeber necessary to the fetus is maternal melatonin, which crosses the placenta rapidly and unaltered to provide photoperiod information [91]. Melatonin receptors are present at 18 weeks of gestation in the fetal SCN and are widely distributed in fetal tissue [92]. The human fetus and neonate do not secrete melatonin, as synthesis begins post-natally [93] and are therefore dependent on maternal melatonin for circadian rhythmicity and development in the absence of external light-dark information. Results from Bellavia and colleagues [94] indicate that post-natal litter synchronization was not present among rat pups born to mothers that had undergone a ganglionectomy and pinealectomy, resulting in a nonfunctional SCN. Synchronization was restored when pregnant rats received melatonin in a diurnal fashion during the late period of gestation, indicating the importance of maternal melatonin in post-natal rhythms. Additionally, under constant maternal light conditions in utero and subsequent constant maternal melatonin suppression, newborn capuchin monkeys had twice as much plasma cortisol compared to those developed under normal light:dark conditions [95]. Those born to mothers who were exposed to chronic light but received daily melatonin replacement displayed normal cortisol levels, suggesting that maternal melatonin serves as an aid in the development of cortisol regulation in fetuses. Collectively, these studies indicate that maternal melatonin serves as a cue for developing fetal circadian rhythms and is important for healthy circadian outcomes.

A modern example of chronodisruption that pregnant women may face is shift work, which disrupts melatonin rhythms, such circadian misalignment also likely impacts fetal circadian development. Epidemiological data indicate that shift work during pregnancy is associated with increased spontaneous abortions, premature delivery, and low birth weight [96–98]. Data from one study in pregnant rats that simulated shift work found long terms effects on dams, including disrupted timing of corticosterone, leptin, glucose, insulin, free fatty acids, triglycerides, and cholesterol concentrations, as well as arrhythmic expression of circadian clock genes in the liver [99]. Further research on the longitudinal effects of gestational chronodisruption is necessary to understand how desynchronized signals during pregnancy impact fetal circadian system development.

### Fetal circadian entrainment through time restricted feeding

Time restricted feeding during pregnancy can serve as a circadian cue to the developing fetus. SCN-lesioned rats produce offspring that display asynchronous litter drinking rhythms; however, restricting the pregnant rat’s feeding schedule to a 4-h window each day resulted in offspring that exhibited synchronization both within and between litters, indicating that prenatal restricted feeding can entrain fetuses [100]. Additionally, when offspring of SCN-lesioned, restricted rats were raised by lesioned, unrestricted mothers, similar synchronicity was observed, supporting the circadian effect of prenatal restricted feeding in utero. Similarly, Nováková et al. [101] found that maternal exposure to constant light during pregnancy, a condition which inhibits time-of-day cues, resulted in flattened circadian rhythmicity of Avp and c-fos gene expression in the SCN of rat pups. However, these rhythms developed normally when pregnant rats were restricted to a 6-h feeding window during their normal resting time. Surprisingly, pregnant rats that were exposed to a LD cycle and restricted feeding during their normal resting time reared pups that were not impacted despite only being able to eat during their rest time. This may be explained by findings from another study that observed maternal feeding during the light portion of a LD 12:12 light cycle entrained the fetal SCN to the maternal feeding time rather than the light schedule that the mother was entrained to, suggesting that the fetal SCN may not be exclusively controlled by the maternal SCN [102]. These studies indicate that rhythmic signals from the mother are necessary for fetal entrainment and there may be a hierarchy of redundant maternal mechanisms (e.g., melatonin, cortisol, food timing) that can serve as time-of-day cues to the developing fetus. However, the interaction and importance of these different maternal signals is not well understood and should be further investigated.

## Factors influencing circadian development in infancy

### Neonatal SCN

At birth, neonates lose direct communication with their mother’s SCN and must develop their own independent oscillating master circadian clock, undergoing a substantial change in function and reorganization of their circadian system. Infants are born with an immature circadian system that does not produce overt rhythms as evident by their absence of significant circadian rhythmicity in melatonin and cortisol rhythms and a stable sleep-wake cycle [39, 93, 103]. The neonatal SCN is not fully developed and contains only 13% of the adult number of AVP-expressing neurons and few VIP-expressing neurons—adult levels are attained by the first 2–3 years of life [82, 104]. During this sensitive developmental window, the SCN may be vulnerable to maternal and environmental influences that impact maturation of the circadian system.

### Light

Light is the strongest environmental time cue to our internal clocks and exposing the developing clock to non-optimal lighting may have disruptive and long-lasting effects. In mice specifically, Ohta and colleagues [105] found that raising pups in constant light for their first 7 weeks of life resulted in disrupted circadian locomotor activities and cellular desynchrony exhibited through weak SCN clock gene oscillations. Conversely, the same researchers observed that mice raised in a LD 12:12 light cycle for their first 3 weeks of life and then exposed to continuous light conditions displayed orderly circadian rhythmicity for several weeks and were less vulnerable to disrupted circadian locomotor patterns [106]. Similarly, another study assigned mice to two different photoperiods (short-day, LD 8:16 or long-day, LD 16:8) during their first 3 weeks of life and then subjected each group to subsequent altered photoperiods for the next 4 weeks. Results indicated that compared to those raised under the short-day condition in very early life, those raised under the long-day condition exhibited a more stable molecular rhythm that tracked closely with the timing of dusk during later exposure to both long-day and short-day cycles [107]. Thus, the first few weeks of life may represent a sensitive window of circadian programming and organization where developing clocks are more susceptible to light exposure, and development during this time may set the stage for responsiveness to altered light cycles later in life.

Little is known about the impact of light on circadian entrainment in primate newborns. In one study, researchers exposed infant baboons to light at 22:30 for 50 min and observed increases in SCN metabolic activity and gene expression [108]. Data from this study also found that these newborns were able to entrain to a low intensity (200 lux) light:dark cycle. These findings indicate that the biological clock of premature infants is responsive to light via the RHT [109]. In humans, the RHT can be detected at 36-week gestation [110], indicating one developed pathway to the SCN by birth. In human neonates, constant dark conditions during the first days of life significantly increased melatonin plasma levels relative to a normal light:dark cycle [111], suggesting that the neonate is sensitive to light exposure post-birth and has the ability to establish connectivity between the SCN and the retina.

The preterm newborn represents a unique clinical context in which direct circadian signals are prematurely lost between the pregnant woman and fetus and replaced with environmental signals through the light-dark cycle. These newborns remain in the hospital for weeks or months where the light schedule is determined by medical personnel. Data from clinical studies indicate that exposing preterm infants to a light:dark cycle rather than constant dim light is associated with distinct patterns of rest and activity that appear 1 week after discharge, better growth rate, reduced fussing and crying, and a shorter hospital stay [112–116]. Additionally, a 24-h sleep-wake rhythm appeared earlier in preterm infants compared to term infants kept under the same cycled light, indicating that external time cues may play a vital role in sleep-wake rhythm development [117]. These studies provide evidence of light detection and entrainment in human preterm infants and highlight the importance of environmental signals for healthy development of the circadian system and sleep.

### Circadian signals in breast milk

In addition to direct environmental circadian signals, infants maintain their connection to the maternal SCN by receiving maternal time cues through breast milk. Data from animal and human studies suggest that the diurnal fluctuations in breast milk evolved to send vital time-of-day information—a form of chrononutrition—to promote the development of an entrained circadian clock [118]. Human milk expressed during the day contains higher levels of cortisol [119], tyrosine [120], and immune factors [121], whereas night milk is comprised of increased levels of leptin [122], melatonin [123], and tryptophan [120]. Lodemore and colleagues [124] found that circadian rhythmicity in body temperature appeared earlier in breastfed infants compared to bottle fed infants, suggesting that breast milk may facilitate maturation of the clock. It is likely that infants reap the benefits when they drink circadian matched breast milk as these hormonal time signals may have evolved to support circadian system development.

### Melatonin

Neonates rely on exogenous melatonin found in breast milk for their nightly hormonal circadian time cue. In humans, breast milk exhibits a congruent circadian melatonin pattern to that detectable in blood and saliva, and the concentration is approximately 35% of that found in the mother’s blood serum [123, 125]. From the maternal pineal, melatonin is secreted into the bloodstream and diffused into the mother’s breast milk [123, 125], where it crosses the infant’s intestinal barrier and circulates into tissues [126]. Here, it serves as a regulator of the infant circadian system. Compared to formula fed infants, breastfed infants show more regular nocturnal increases in 6-sulfatoxymelatonin, a metabolite of melatonin excreted in urine [127], higher sleep efficiency, less fragmented sleep [118], longer nocturnal sleep duration, and lower incidence of infant colic [125]. In the absence of breast milk, supplementation with exogenous melatonin may promote circadian clock development; however, this research remains unexplored. Infants given tryptophan-enriched formula, a melatonin metabolite precursor, displayed improvements in sleep and increases in urinary metabolites of serotonin from internal serotonin to melatonin synthesis [128]. Injection of melatonin in neonatal hamsters born to SCN-lesioned mothers resulted in entrained circadian rhythms of wheel-running activity on the day of weaning, PND21 [129]. Cumulatively, these data suggest that exogenous forms of melatonin serve as early circadian signals to the developing clock and are a necessary component to facilitate entrainment to the day/night cycle.

### Cortisol

The circadian rhythm of cortisol in infants has been observed from as early as 2 weeks up to 9 months of age and is linked with the emergence of the sleep-wake cycle [103, 130–132]. The wide range in the cortisol rhythm appearance may be due to varying exposures of exogenous cortisol through breast milk. In humans, higher milk cortisol levels are associated with developmental outcomes including lower BMI and higher fear reactivity [133, 134]. Similar to maternal melatonin, cortisol in breast milk circulates from the maternal plasma and follows the same diurnal variation found in plasma and saliva [119, 135]. Human milk cortisol levels vary based on season and psychological distress [136, 137]. Data from rodent studies have established that ingested glucocorticoids diffuse from the neonatal intestines into the plasma and brain [138]. In adults, cortisol has been established as a major entrainment factor for peripheral clocks, indicating a pathway by which milk cortisol may regulate the infant peripheral clocks [59].

## Summary and future directions

In the context of post-modern electric lighting environments, pregnant women and infants face daily challenges of circadian disruption, and the associated early developmental outcomes remain poorly understood. In a case study of an infant who was breastfed and was only exposed to natural light for the first 6 months of life, measurable outputs of circadian rhythmicity appeared more rapidly than previously reported—the temperature rhythm was observed at 1 week of age, rhythms of wake and melatonin secretion at sunset emerged at day 45, and nighttime sleep onset aligned with sunset by day 60 [139]. This finding suggests that infants growing in the natural world with no electric lighting results in more favorable circadian-related developmental outcomes. Longitudinal studies to determine how maternal and environmental signals interact with circadian system development, such as breast milk and the light/dark cycle (Fig. 1) are necessary to establish what circadian cues to prioritize in prenatal and post-natal maturation.

Research in the field of circadian rhythm development has centered on two developmental timepoints, the prenatal and post-natal periods. As previously discussed, rhythms in the prenatal stage are influenced by maternal signals while rhythms in the post-natal stage are guided by environmental signals that the infant is exposed to as well as a maternal connection via breast milk. Although these two periods are distinct, they should not be treated as unconnected—prenatal development likely sets the stage for healthy post-natal development. Few studies have followed women and fetuses from pregnancy through early development in post-natal life. Capitalizing on such longitudinal data would allow examination of how factors during pregnancy may impact later development.

Although the multi-dimensional construct of “sleep health” as measured by self-reports and actigraphy has received considerable attention in the past decade [140–142], “circadian health” is a construct that is presently not well-defined, especially in the context of development. In part, this may be due to the demands of reliable measurement of circadian outputs, such as cortisol, melatonin, and core body temperature, but also reflects less developed conceptualization of a full distribution of core behavioral and physiological circadian features from good to poor. The SCN is complex with multiple neuronal and glial cell types, as well as different subregions and outputs. Therefore, within the SCN there is likely not a single biomarker that would reflect good circadian health. Understanding of how the SCN communicates with peripheral clocks is rapidly advancing based upon animal and adult human data; however, a similar appreciation is limited across development. Indeed, a significant component of circadian health may be at the level of communication between the SCN and peripheral clocks through entrainment signals. Additionally, most circadian research centers on how circadian systems respond to perturbation rather than how to create optimal environments for good circadian health. Also, current studies focus on the time course of clock outputs to quantify circadian clock development; however, with few longitudinal studies available, it is difficult to determine if early rhythms are sensitive predictors of an adaptive system later in life. Other features indicating optimal circadian system maturation may include robustness of rhythms, adaptability of the clock to changing environments, and consistency of rhythms. Future developmental research should consider not only clock outputs but also such metrics in early life that may predict circadian health across the lifespan.

Pregnant women, mothers, and other infant caregivers serve as gatekeepers to external factors that influence circadian system development. Conventional behaviors in our post-modern world can result in conflicting temporal time cues for developing fetuses and infants. During pregnancy, a woman’s physiological signals set time-of-day cues for the fetus, and exposure to light at night results in a desynchronized circadian system that may disorganize the fetus’ circadian development and adaptation to environmental stimuli. Environmental lighting may also impact the developing infant through direct and indirect signals. For example, infants sleeping in a darkened room during daytime naps or exposed to light at night during feedings may experience a misperception of the time-of-day. Also, mothers breastfeeding at night may turn on an electric light or use a blue light-enriched device, which likely suppresses melatonin and other nighttime hormones found in breast milk, thus dampening maternal time cues to the infant’s circadian clock. Further, data suggest that infants who are fed formula do not benefit from the diurnal fluctuation of hormones present in breast milk. Additionally, women who pump may record the date but not the time-of-day that milk was expressed, which can result in infants receiving milk at a non-optimal circadian time. That is, if a woman feeds her infant milk in the evening that she previously pumped during the day, the infant will not receive nighttime hormonal time cues such as melatonin. The outcome of these mismatched signals and how they interact in early development and beyond remain unknown. One important question illustrating this type of interaction is if an infant is fed milk containing melatonin during the night but is exposed to artificial light. Which time cue is dominant? Or what if milk for a nighttime feeding originates from daytime expressed milk and the infant is also exposed to light—how do these factors synergize? Understanding such interactions will inform early prevention and intervention programs on modifiable factors that influence circadian system maturation, which could be cost effective and easily implemented. Based upon the above reviewed literature and the importance of the circadian system for health and development, future work should focus on the extent to which conflicting time cues influence early clock development and propose evidence-based parental care habits that will strengthen the daily environmental synchronization of the developing human.

## Data Availability

Not applicable

## Abbreviations

ACTH: Adrenocorticotropic hormone
AVP: Arginine vasopressin
Bmal1: Brain and muscle Arnt-like factor
CAR: Cortisol awakening response
Clock: Circadian locomotor output cycle kaput
GAPVD1: GTPase activating protein and VPS9 domains 1
LD: Light:dark
PND: Post-natal day
Rev-Erbα: Reverse erythroblastosis virus α
RHT: Retinohypothalamic tract
Rorα: Retinoic acid receptor-related orphan receptor α
SCG: Superior cervical ganglion
SCN: Suprachiasmatic nucleus
VIP: Vasoactive intestinal polypeptide
